# Chronic Immune Sensory Polyradiculopathy (CISP): A Systematic Review of the Literature

**DOI:** 10.3390/neurolint16060092

**Published:** 2024-10-25

**Authors:** Saurabh Singhal, Rahul Khanna, Anudeep Surendranath, Jayksh Chhabra, Vismay Thakkar, Rajesh Gupta

**Affiliations:** 1 Department of Neurology, Well Smart Health Neurology Clinic, Opelousas, LA 70570, USA; 2 Neurology and Sleep Clinic, PLC, West Burlington, IA 52655, USA; rahulkhanna.doctor@gmail.com; 3 Neurology, CHI St. Vincent, Hot Springs, AR 71913, USA; 4 Department of Neurology, University of Texas Health Science Center, Houston, TX 77030, USArajesh.k.gupta@uth.tmc.edu (R.G.); 5 Neurology, Wesley Medical Center, Wichita, KS 67214, USA

**Keywords:** CISP, chronic immune sensory polyradiculopathy, CIDP, AIDP, ataxia, SSEP

## Abstract

Chronic immune sensory polyradiculopathy (CISP) is a rare inflammatory immune disorder affecting the nervous system, primarily targeting the proximal sensory nerve roots. The condition was first described by Sinreich in 2004. We conducted a systematic review of CISP cases published on PubMed to identify common clinical presentations, along with neurophysiological, radiological, cerebrospinal fluid (CSF), and other findings. Our review included a total of 22 patients from 8 articles. Many patients presented with gait difficulties and sensory ataxia and were found to have normal nerve conduction studies (NCS) and electromyography (EMG) but exhibited characteristic abnormalities in somatosensory evoked potentials (SSEP), elevated CSF protein levels, thickened nerve roots on contrast-enhanced lumbar spine MRIs, and histological changes on nerve root biopsies. Clinical improvement was observed following treatment with steroids and/or intravenous immunoglobulin (IVIG). The study concluded that while CISP is rare, it is an important clinical entity to consider, as accurate diagnosis and appropriate treatment can lead to significant improvements in neurological symptoms and disabilities.

## 1. Introduction

Chronic immune sensory polyradiculopathy (CISP) is a rare and often overlooked inflammatory/immune nervous disorder. It is characterised by the isolated involvement of the dorsal roots proximal to the dorsal root ganglion, typically sparing the distal nerve roots, distinguishing it from more commonly known conditions such as acute inflammatory demyelinating polyneuropathy (AIDP) and chronic inflammatory demyelinating polyneuropathy (CIDP) [1]. First identified by Sinreich et al. in 2004 [1], CISP is typically marked by large fibre sensory loss, sensory gait ataxia, areflexia/hyporeflexia, normal motor examination, normal nerve conduction study (NCS)/electromyography (EMG) electrodiagnostic (EDX) studies, distinctive somatosensory evoked potential (SSEP) abnormalities, elevated protein levels in cerebrospinal fluid (CSF) from lumbar puncture, thickening of lumbar nerve roots on magnetic resonance imaging (MRI), inflammatory demyelinating changes on lumbar root biopsies, and improvement in symptoms with immune-modulating treatment [1,2]. The diagnosis is often missed due to normal EDX and CNS imaging findings leading to patients’ clinical presentation being incorrectly labelled as hysterical or non-organic.

To the best of our knowledge, there has not been any recent significant study to inform us about the incidence of the disease and the prevalence of characteristic presenting symptoms or clinical and diagnostic findings with treatment outcomes. Here, we conducted a systematic literature review of the CISP cases that presented with characteristic neurological symptoms, with clinical, radiological, neurophysiological, CSF, and other findings leading to an accurate diagnosis followed by treatment with improvement in the clinical condition.

## 2. Methods

**Figure 1:**
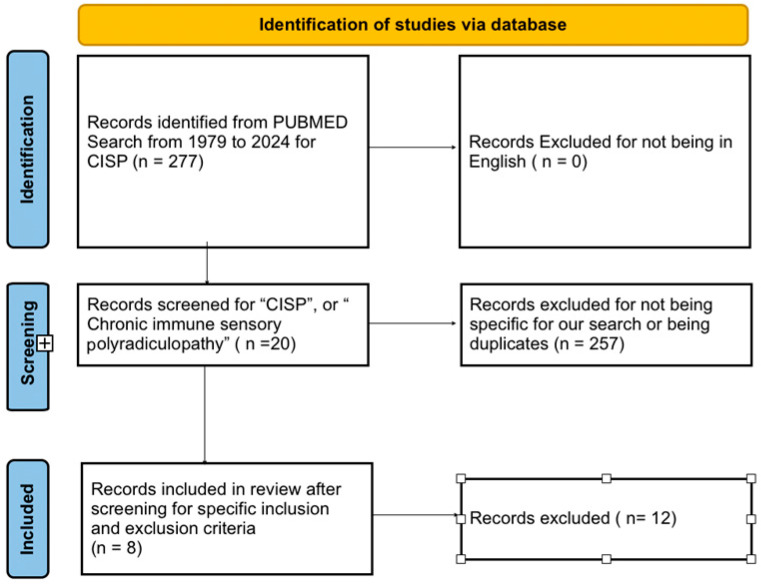
PRISMA table for the literature review.

We registered our systematic review with PROSPERO (Registration ID: CRD42024580663). We followed the “Preferred Reporting Items for Systematic Reviews and Meta-Analyses (PRISMA)” guidelines for our systematic review. We conducted literature searches in Pubmed (Figure 1) using the keywords for “CISP”, or “Chronic immune sensory polyradiculopathy”. The initial search was filtered to show articles from 1 st January 1979 to 17 th June 2024. We removed duplicates from our search results. We filtered the results to show case reports and case series. We removed case reports fulfilling the European Federation of Neurological Societies (EFNS) criteria for diagnosis of classic CIDP; those involving combined sensory and motor involvement (CISMP), pure motor involvement (CIMP), sensory variant of CIDP, non-pure “CISP plus” cases, sensory neuronopathy (paraneoplastic and Sjogren syndrome), or known causes of neuropathy such as vitamin deficiency, diabetes, alcoholism, thyroid disease, hereditary neuropathies, metal intoxication, uremia, etc., We found 8 articles with a total of 22 patients that were eligible for our systemic review. The specific inclusion and exclusion criteria used are detailed below.

Inclusion criteria included articles with patients satisfying the specific diagnostic criteria for CISP for localised neuropathy of the posterior roots: (1) presence of a sensory syndrome without associated weakness; (2) normal or near-normal nerve conduction and EMG studies; (3) imaging studies ruling out lesions in the brain, cerebellum, spinal cord, or compressive nerve root pathology; and/or (4) abnormalities in either SSEP or imaging indicative of nerve root involvement. Exclusion criteria were used to specifically focus the review study for the diagnosis of CISP and remove all other related/overlapping clinical variants. This was to avoid variability and maintain homogeneity among the cases selected, as well as to narrow the focus of the review strictly to the diagnosis of CISP. The exclusion criteria included patients involving mixed motor symptoms and/or later diagnosed with variants such as CISMP, CIMP, sensory variant of CIDP, sensory neuronopathies, etc. We also excluded articles published in non-English languages and articles that republished previously reported cases. The specific inclusion and exclusion criteria helped minimise selection bias.

We carefully evaluated each article for descriptions of characteristic neurological presenting symptoms, age, sex, duration of symptoms, clinical neurological exam, CSF protein level, radiological results, EDX results, SSEP results, quantitative sensory testing and lumbar nerve root biopsies, and finally, treatments and outcomes. We confirmed that all case reports met high quality standards based on “The Joanna Briggs Institute (JBI) Critical Appraisal Tools for use in JBI Systematic Reviews”. Each article was scrutinised per JBI Critical Appraisal Checklist independently by the lead author (S.S) and the last author and supervisor of the project (R.G). This further helped reduce selection bias. We interpreted continuous variables as the mean with standard deviation with a margin of interval for 95% confidence level, and categorical variables as frequencies, and percentages.

## 3. Results

We reviewed case reports and one short case series of CISP patients who presented with the characteristic features as above and found a total of 22 patients in 8 eligible articles. We analysed the data of these 22 patients. Demographic and clinical data are presented in Table 1.

Our cohort had predominantly male patients: 7 out of 20 patients were female (35%), while 13 were male (65%). No data were available regarding sex in the remaining two patients.

Mean presenting age ranged from 17 years to 78 years in 21 patients, with a mean of 52.8 years with std deviation of 17.07 (no data available for one patient), and a margin of interval for 95% confidence level of 52.38 +/− 7.301.

Duration of symptoms on presentation ranged from 2 weeks to 18 years in 21 patients, with a mean of 4.912 years, std deviation 4.89, and a margin of interval for 95% confidence level of 4.912 +/− 2.09 years.

14 out of 20 patients (70%) presented with ataxia requiring gait aids and 64.7% presented with falls. Of 21 patients, all 100% had large fibre sensory loss in the lower extremity, 42.86% had similar loss in upper extremity, 90% had reduced or absent reflexes in lower extremity while 62% had similar in upper extremity, signifying the predominance of lower extremity involvement with mainly the large fibre nerve impairment. None of the patients (0%) had weakness.

The neuropathy impairment score (NIS) was available for 15 patients, with values ranging from 10 to 49 and a mean of 25.33 with a standard deviation of 10.86 and margin of interval for 95% confidence level of 25.33 +/− 5.496.

Diagnostic Testing and Treatment are presented in Table 2. Protein level in CSF on LP was available in 21 out of 22 patients’ data. Out of these, 20 patients had elevated CSF protein (normal CSF protein <45 mg/dL), representing 95% of patients (20 out of 21). Patients’ CSF protein levels ranged from 30 to 350 mg/dL with a mean of 105.3 mg/dL, with a standard deviation of 64.51 and a margin of interval for 95% confidence level of 105.3 +/− 28.273.

Lumbar spine MRI showed thickening of nerve roots in 9 out of 22 patients (41%). One patient had normal lumbar spine nerve roots, but had enlargement of right brachial plexus, roots, and trunks with mild contrast enhancement of roots of the same. One patient had an MRI performed after IVIG, and one patient only had a non-contrast MRI. 15 patients had data available showing normal MRI imaging of the brain, cerebellum, and spinal cord.

For the data available on NCS/EMG findings, 19/19 patients (100%) had normal sural nerve findings, 15/15 patients (100%) had normal tibial nerve amplitude, conduction velocity and distal latency. Tibial F waves were studied in 5 patients, of which 3 had prolonged (60%) and 2 absent (40%), and overall, all 5 patients (100%) presented abnormally.

Tibial H reflexes were absent in all 3/3 patients reported (100%). Needle EMG of extremities was normal in all except patient #6, shown in an old S1 radiculopathy.

For the SSEP, Tibial SSEP slowing/prolongation/abnormality were noted in 16/19 patients (84%), whereas Median SSEP slowing/prolongation/abnormality were noted in 8/15 patients (53.33%), signifying the importance of SSEP in lower extremity as a potential diagnostic tool when presented with normal NCS/EMG values.

For the Quantitative Sensory Testing

(1) VDT % were available in all 11/11 patients, of which all were high (100%); these are mediated by large Aαβ fibres signifying predominant involvement of the large fibres.

(2) CDT % were elevated in 3 out of 11 patients (27%); these are mediated by small myelinated Aδ fibres.

(3) HP5 % was elevated, among the 10 patients with data available, in 2 patients, and 2 had reduced Heat Pain 5 levels mediated by unmyelinated C fibres.

For the treatment response, we obtained data concerning treatment in a total of 14 patients. A total of 4 patients were treated with prednisone with improvement in 2 patients. The other 2 patients did not respond, requiring treatment with IVIG, one of which was also treated with mycophenolate mofetil in combination with IVIG; both of them showed improvement. A total of 8 patients were treated directly with IVIG, with improvement/response recorded in 6 patients, and no data available for the remaining two patients. Additionally, 1 patient was treated with IV methylprednisolone and 1 with iv dexamethasone both with clinical improvement.

## 4. Discussion

CISP is a very uncommon but treatable inflammatory/immune disorder of the proximal sensory nerve roots. It can be easily missed given normal routine electrodiagnostic and imaging findings. However, this can be avoided by paying careful attention to the characteristic features of the clinical presentation, neurological exam, and obtaining further workups such as CSF studies, MR with contrast imaging of the lumbar nerve roots, SSEP, biopsies of the nerve roots, or quantitative sensory testing. Appropriate diagnosis and treatment can result in significant improvement in the presenting symptoms and neurological deficits [1,2,3,4,5,6]. Because of the rarity of the disease, there are limited publications and case reports on this in the literature. Here, we performed a systemic literature review of the CISP cases reported and analysed the aforementioned common characteristic features of these.

Patients with CISP typically present with gait imbalance, sensory ataxia, and falls. The mean duration of presentation from symptom onset was 4.9 years in our review. Our review also showed that 67% patients presented with ataxia and 64.7% had falls. Patients typically (1) have large fibre sensory loss in lower extremities such as seen in 100% of our patients, (2) reduced or absent reflexes in lower extremities (95% of our patients), and (3) often the upper extremities are involved but not as much as the lower extremities–our review had large fibre sensory loss of upper extremities in 42.86% and impaired UE reflexes in 60% patients. None of our 22 patients (0%) presented with weakness.

The neuropathy impairment score (NIS) was used to objectively assess the progression of neuropathic symptoms in 15 of our patients. NIS is a clinician-related measure with a subscale score for sensory loss ranging from 0 to 32. The higher the score, the greater the neuropathic impairment [7]. The mean nerve impairment score (NIS) in our review was 25.33. Stefano et al. [3] used an INCAT-ODSS score to assess the clinical presentation of the first juvenile case of CISP at age 17 yrs; the score was reported to be of 4 (range 0–12). This was also used by Clerici et al. [4] in their case report of a 30 year old woman who presented with an ODSS score of 4.

CISP patients typically have normal NCS findings due to the selective involvement of proximal sensory nerve roots, while sparing the distal nerves. In our review, 100% of patients had normal sural and tibial nerve amplitudes, conduction velocity, and latencies. Tibial nerve late responses (F waves and H reflexes) data were available in 8 patients and they were all abnormal, indicating proximal nerve root involvement. Our review had tibial nerve SSEP abnormality in 84%, signifying proximal nerve root involvement and the importance of SSEPs in diagnosing this rare condition. Median nerve SSEPs were abnormal in 53.33% of patients. Our review also had CSF LP protein elevation in 95%, which goes along with the important diagnostic criteria for the disorder.

As for the radiological images, while the routine MRI scans of the brain, cerebellum, and spine can be normal, a contrasted study of the lumbar spine MRI may demonstrate nerve root enhancement (as was the case in 41% of patients reported in our review). Diffuse enhancement of cauda equina can be seen as GBS, sarcoidosis, tuberculosis, lymphoma, disseminated metastasis, and hereditary neuropathies; however, they can be differentiated from serum biochemistries, CSF studies, significant response to immunotherapy, and overall course of illnesses [8,9,10].

The Quantitative Sensory Results available in 11 patients demonstrated preferential involvement of large, myelinated nerve fibres. This was further corroborated by the nerve root biopsy results in three patients described by Sinreich et al. in their original paper [1], which showed marked reductions in large myelinated nerve fibres. Other features included lymphocytes (CD45) and macrophages (CD 68) in the endoneurium along with the conspicuous onion bulb formations and myelin remodelling.

Workup performed to rule out other differentials included negative blood work for glucose, lipid profile, TSH, vitamins E, B12, folate, ACE, syphilis, HIV, Hepatitis, copper, serum protein electrophoresis with immunofixation, ceruloplasmin, SCA panel, ENA/ANA/ANCA panel, CSF oligoclonal bands, paraneoplastic panel, GM1 and GD1b antibodies, GQ1b Ab, and MAG Abs [1,2,3,4,5].

There were a total of 14 patients with data available pertaining to treatment modalities. Six were treated with steroids with improvement in four. The other two who did not improve were additionally treated with IVIG with clinical improvement, and one of these also received further treatment with mycophenolate mofetil. Eight additional patients were treated with IVIG with response to treatment noted in six of these patients, while no data available in terms of response in the remaining two patients. Responses were measured with clinical improvement in ataxia and ambulation, as well as improvement in reflexes and sensations. Overall, these indicate favourable responses to immunotherapy and further signify the demyelinating nature of the radiculopathy.

Koh et al. [2] described the case of a 80 year old male patient who presented acutely within a couple of weeks requiring aggressive combination treatment with steroids, IVIG, mycophenolate mofetil, unlike most other CISP patients presenting months or years after symptom onset and improving after steroids and/or IVIG. They suggested early diagnosis with aggressive treatment with combined immunotherapies before the Wallerian degeneration is advanced in the spinal nerve roots. They also suggested follow-up SSEPs along with clinical functional scores such as mRS to measure response to treatments. The use of SSEPs in monitoring the response of IVIG treatment was also mentioned by Clerici et al. [4] who additionally used the clinical INCAT-Overall Disability sum score allowing modulation between IVIG cycles, with a significant reduction in clinical fluctuations and disability.

Sinreich et al. [1] also mentioned other variants of CIDP such as classic CIDP presenting with symmetric proximal and distal motor predominant symptoms; MMN (multifocal motor neuropathy) with only motor symptoms but multifocal; paraprotein associated CIDP with distal sensory predominance similar to distal acquired demyelinating symmetric neuropathy (DADS); Lewis-Sumner syndrome (LSS) with multifocal sensorimotor; chronic sensory demyelinating neuropathy (pure sensory variant of CIDP) [11] with purely sensory symptoms such as paranoidal neuropathies (contactin-1-associated CIDP) [12,13]. The last one differs from CISP in that the former would involve distal sensory nerves with abnormal NCS, which are spared in CISP cases. Stefano et al. [3], who described the first juvenile case of CISP (age 17 years), mentioned pure sensory DADS and pure sensory LSS. Also cited were retrospective analyses from a large Italian database that described atypical variants of CIDP in up to 39% of cases; with CISP accounting for only up to 0.5% of all the diagnoses [14]. Shelly et al. [15] further described rare non-pure cases of CISP as “CISP plus” involving motor and distal sensory nerves unlike classic CISP, but otherwise having elevated CSF protein; slowing of SSEPs; MRI nerve root enhancement; loss of large fibre myelinated nerve fibres; onion bulb formations predominantly in rootlet biopsies; and improvement with immunotherapy, similar to the classic CISP patients. They also reported autonomic reflex screens in 10 patients with pure CISP with a median composite autonomic scoring scale score of 0 (range 0–4) indicating mild autonomic involvement.

The pure motor counterpart of CISP, termed as CIMP, has been reported [8] with a single case described as a patient with gradually progressive bilateral lower extremity weakness and back pain without any sensory deficits. Khadilkar et al. coined the term CISMP [16], with two patients having both sensory ataxia and weakness (unlike the pure sensory symptoms of CISP), however having other characteristics similar to CISP with proximal nerve root involvement, lack of demyelination on NCS, thickened nerve roots on MRI, and elevated CSF protein. CISMP differs from CISP plus in that CISMP cases present with predominantly weakness (particularly of the lower extremity) and less frequently sensory ataxia, while all of the CISP plus patients have sensory ataxia as the main presenting symptom with minimal weakness. Once again, weakness is universally absent in the classic CISP cases. Khadilkar and colleagues also suggested the use of terms CISMP, CIS(m)P, and CI(s)MP to demonstrate the predominant clinical involvement. Trip et al_._ [5] described a case of CISP with cranial nerve involvement (oculomotor nerve palsy). They differentiated their case from Miller Fisher Syndrome by the difference in phenotype and negative GQ1 Ab. Kim et al_._ [17] described a case of acute immune sensory dominant polyradiculopathy predominantly involving thoracic nerve roots.

## 5. Limitations

The primary limitation of our study is the small sample size of cases reviewed. The sample size of 22 patients from 8 articles is notably small, primarily due to the rarity of the disease and the limited availability of publications and case reports in the literature. This small sample size reduces the statistical power of the study, potentially limiting the generalizability of the findings to the broader population of CISP patients. As a result, the study may not capture the full spectrum of the disease process or responses to treatment. Nevertheless, this remains the first systematic review of this rare condition, and we hope it will assist neurologists in better identifying and documenting this clinical entity, leading to larger-scale studies in the future. Additional multi-centre studies with larger sample sizes will be necessary to validate the findings of this review.

## 6. Conclusions

The review study concluded that, despite its rarity, CISP is a crucial clinical condition to consider, as proper diagnosis and treatment can lead to substantial improvements in neurological symptoms and disabilities. The typical clinical presentation includes large fibre pure sensory loss without associated weakness and sensory gait ataxia with a normal motor examination. Common diagnostic findings are normal NCS/EMG studies, characteristic SSEP abnormalities, elevated protein levels in the CSF from lumbar puncture, thickening of lumbar nerve roots on MRI, inflammatory demyelinating changes on lumbar root biopsies, and symptom improvement with immune-modulating therapy. While the study is limited by the small number of clinical cases currently reported in the literature so far, it is hoped that our study will aid in preventing missed diagnoses in the future, encouraging the publication of more case reports and larger studies to further confirm these findings.

## Figures and Tables

**Table 1:** Demographics, Symptoms and Examination.

Charactestics	Number/Mean	Range/Percentage
Male	13/20	65%
Female	7/20	35%
Presenting age	Mean 52.8 years	17 to 78 years
Duration	Mean 4.912 years	2 weeks to 18 years
**Symptoms and Examination**	**Frequency (case count)**	**Frequency (%)**
Sensory Ataxia	14/20	70%
Frequent falls	11/17	64.70%
Large fibre sensory loss-UE	9/21	42.86%
Large fibre sensory loss-LE	21/21	100%
Small fibre sensory loss-UE	8/21	38%
Small fibre sensory loss-LE	13/21	62%
Reflexes-UE	13/21	62%
Reflexes-LE	19/21	90%
NIS	Mean-25.33	range-10–49

**Table 2:** Diagnostic Testing and Treatment.

Diagnostic Features/Characteristics	Number	Range/Percentage
Elevated CSF protein	20/21	95%
Thickened lumbar root on MRI	9/22	41%
Tibial SSEPabnormality	16/19	84%
Median SSEP abnormality	8/15	53%
**Quantitative Sensory Testing**		
VDT %	11/11	100%
CDT %	3/11	27%
**NCS/EMG FINDINGS**		
Normal Sural Sensory Response	19/19	100%
Normal Tibial Motor Response	15/15	100%
Abnormal Tibial Late Responses	5/5	100%
**Treatment**		
IVIG	8/14	57%
Steroid	6/14	43%
Others	2/14	14%
**Positive response to treatment**		
IVIG	8/8	100%
Steroid	4/6	67%
others	2/2	100%

## Data Availability

The original contributions presented in the study are included in the article/references listed below, further inquiries can be directed to the corresponding author/s.
